# Intronic variants in inborn errors of metabolism: Beyond the exome

**DOI:** 10.3389/fgene.2022.1031495

**Published:** 2022-12-06

**Authors:** Ashley Hertzog, Arthavan Selvanathan, Elizabeth Farnsworth, Michel Tchan, Louisa Adams, Katherine Lewis, Adviye Ayper Tolun, Bruce Bennetts, Gladys Ho, Kaustuv Bhattacharya

**Affiliations:** ^1^ NSW Biochemical Genetics Service, Western Sydney Genetics Program, The Children’s Hospital at Westmead, Sydney, NSW, Australia; ^2^ Disciplines of Genetic Medicine and Child and Adolescent Health, Faculty of Medicine and Health, University of Sydney, Sydney, NSW, Australia; ^3^ Genetic Metabolic Disorders Service, Sydney Children’s Hospitals Network, Sydney, NSW, Australia; ^4^ Department of Molecular Genetics, Sydney Genome Diagnostics, Western Sydney Genetics Program, The Children’s Hospital at Westmead, Sydney, NSW, Australia; ^5^ Department of Genetic Medicine, Westmead Hospital, Sydney, NSW, Australia

**Keywords:** inborn error of metabolism, intronic variant, non-coding variant, genotype-phenotype correlation, promoter variant

## Abstract

Non-coding regions are areas of the genome that do not directly encode protein and were initially thought to be of little biological relevance. However, subsequent identification of pathogenic variants in these regions indicates there are exceptions to this assertion. With the increasing availability of next generation sequencing, variants in non-coding regions are often considered when no causative exonic changes have been identified. There is still a lack of understanding of normal human variation in non-coding areas. As a result, potentially pathogenic non-coding variants are initially classified as variants of uncertain significance or are even overlooked during genomic analysis. In most cases where the phenotype is non-specific, clinical suspicion is not sufficient to warrant further exploration of these changes, partly due to the magnitude of non-coding variants identified. In contrast, inborn errors of metabolism (IEMs) are one group of genetic disorders where there is often high phenotypic specificity. The clinical and biochemical features seen often result in a narrow list of diagnostic possibilities. In this context, there have been numerous cases in which suspicion of a particular IEM led to the discovery of a variant in a non-coding region. We present four patients with IEMs where the molecular aetiology was identified within non-coding regions. Confirmation of the molecular diagnosis is often aided by the clinical and biochemical specificity associated with IEMs. Whilst the clinical severity associated with a non-coding variant can be difficult to predict, obtaining a molecular diagnosis is crucial as it ends diagnostic odysseys and assists in management.

## Introduction

The human genome comprises three billion base pairs of deoxyribonucleic acid (DNA), and only 2% of these bases directly encode for protein. The remaining 98% of the genome (including introns, regulatory elements and intergenic sequences) was originally thought to be “junk DNA” (Ohno, 1972). However, it has become apparent that larger non-coding regions distinguish complex eukaryotes from prokaryotes (Belshaw and Bensasson, 2006), suggesting that many of these regions must have some physiological role. In 1987, Walter Gilbert suggested through his ‘intron-early’ theory that introns played a role in formation of modern genes, by allowing for restructuring of mini-exons that later formed a gene (Gilbert, 1987). However, there have been additional roles subsequently identified, including regulation of transcription initiation and termination, and genomic organization (Chorev and Carmel, 2012).

Many mechanisms by which non-coding variants can be responsible for disease have been established and comprehensively summarized by Ellingford and colleagues (Ellingford et al., 2022). However, due to limited population-wide whole genome sequencing (WGS) data, there is a lack of understanding of normal human variation in these non-coding areas. In most cases where the phenotype is non-specific, the clinical index of suspicion is not strong enough to warrant further exploration of non-coding changes, partly due to the large number of variants identified.

In contrast, inborn errors of metabolism (IEMs) often have highly specific phenotypes. The clinical and (in particular) biochemical features aid in differentiating individual disorders, narrowing the list of diagnostic possibilities. In this context, there have been numerous cases where high clinical suspicion of a particular IEM led to the discovery of a variant in a non-coding region (Perez et al., 2010). Moreover, variants identified in genes associated with IEMs are more likely to be classified as likely pathogenic or pathogenic, as there is often supportive biochemical evidence. Identifying a non-coding variant in a patient with an IEM is of substantial utility, as it confirms a molecular diagnosis, assists with reproductive planning, and may have management implications.

We present four illustrative cases of patients with IEMs where a molecular aetiology was identified within non-coding regions. The molecular diagnoses were only possible because of a pre-existing strong biochemical suspicion, which influenced variant classification.

## Methods

All patients with reportable intronic variants (those that were classified by the laboratory as likely pathogenic or pathogenic and were potentially disease causing based on zygosity) were compiled from an in-house massively parallel sequencing database (the Children’s Hospital at Westmead Molecular Genetics Department). Whole exome sequencing (SureSelect CREv2, Agilent Technologies, with sequencing on NovaSeq6000, Illumina) was performed to an average read depth of 100x, and only variants in the genes requested by clinicians were analysed. The subset of these patients with IEMs was ascertained by filtering using the PanelApp ‘Inborn Errors of Metabolism Superpanel’ (Stark et al., 2021). Five patients were identified: however, one patient was excluded as they had been previously published (Sajeev et al., 2021).

## Results

### Patient 1: 6-pyruvoyl-tetrahydropterin synthase deficiency

Patient 1, detected by newborn screening (NBS), has hyperphenylalaninaemia (244 **→** 692 μmol/L), which was strongly responsive to BH4 supplementation (92% response). She had a normal newborn examination. Urinary pterin analysis demonstrated a low urine biopterin (0.49 μmol/mmol creatinine) and biopterin/neopterin ratio (1.9%). Urinary 5-hydroxyindoleacetic acid and homovanillic acid were normal, though CSF studies were unable to be performed due to failed lumbar puncture. Neurotransmitter supplementation (with 5-hydroxytryptophan and L-DOPA) was commenced, along with sapropterin. Molecular analysis identified bi-allelic variants (c.286G>A and c.84-291A>G) in *PTS*, consistent with the biochemistry and a diagnosis of 6-pyruvoyl-tetrahydropterin synthase deficiency. The former is a missense variant, and the latter is a deep intronic variant frequently observed in Han Chinese populations (Chiu et al., 2012); both are predicted to result in residual enzyme activity.

Patient 1 is now 2 years old. All neurotransmitters were ceased at around 6 months of age, with no clinical deterioration. She continues on sapropterin, is growing well (74th centile for height, 16th centile for weight) and is making good developmental progress.

### Patient 2: Primary carnitine deficiency

Patient 2 is a 38-year-old woman who was incidentally identified after her first-born child was noted to have low dried blood spot acylcarnitine levels on NBS. Her baby’s plasma acylcarnitine concentrations were normal. Patient 2 had no medical history or current muscle symptoms. Plasma acylcarnitine analysis demonstrated low total (5 μmol/L; RR 21-70), free (5 μmol/L; RR 13-56), and acetylcarnitine (0 μmol/L; RR 3-23) concentrations. Carnitine transport activity in the patient’s skin fibroblasts was markedly reduced (3% of controls). She remains clinically well, and had a normal cardiac review. She was commenced on carnitine supplementation.

Initially, DNA sequencing of *SLC22A5* identified two variants; c.424G>T and c.1463G>A. These two variants have been established as a complex pathogenic allele when in *cis* (Amat di San Filippo et al., 2003); however no other disease-causing variant was identified. Following a subsequent publication by Ferdinandusse and colleagues (Ferdinandusse et al., 2019), the genomic data was re-interrogated for the c.-149G>A promoter variant, which was successfully identified. Segregation studies have been recommended, but could not be performed in this family.

### Patient 3: Fructose-1,6-bisphosphatase deficiency

Patient 3 is a seven-year-old girl and the first child of consanguineous (first cousin) parents. She initially presented to the local Emergency Department with a 24-h history of malaise, abdominal pain, and vomiting. She, as well as her younger brother, had a history of multiple similar episodes. On presentation she was lethargic and hypoglycaemic (1.6 mmol/L; RR 3.5–5.5), with a metabolic acidosis (pH 7.12, base excess -21, lactate 11.8 mmol/L; RR 0–1.9). Urine organic acid analysis showed gross lactic aciduria and ketonuria, with elevations of glycerol and glycerol phosphate. She responded well to intravenous dextrose therapy and was discharged.

Massively parallel sequencing of genes associated with hypoglycaemia identified a previously reported (Emecen Sanli et al., 2022) homozygous likely pathogenic variant (c.705 + 5G>A) in *FBP1*, confirming a diagnosis of fructose-1,6-bisphosphatase deficiency. Segregation studies demonstrated an identical genotype in her younger brother. They are both managed by increasing carbohydrate intake when unwell.

### Patient 4: Hyperphenylalaninaemia

Patient 4 was identified with hyperphenylalaninaemia (HP) on NBS (303 μmol/L and 248 μmol/L; RR < 150). She has a paternal uncle with HP, in the context of multiple loops of consanguinity in the broader family pedigree. Antenatal ultrasounds identified a cystic kidney, which had involuted on post-natal follow-up. Despite having a solitary kidney, she maintained normal renal function. She developed acquired microcephaly (z-score -2.29) and she has marked global developmental delay. Chromosome microarray and fragile X testing were non-contributory. Molecular analysis of a bioinformatic panel of genes associated with increased phenylalanine levels identified bi-allelic pathogenic variants in *PAH*: a likely pathogenic missense variant (c.355C>T) and a pathogenic intronic variant (c.969 + 5 G>A). Phase confirmation of these variants is pending, though they are expected to be in *trans* given the HP. The c.969 + 5G>A variant is predicted to result in a mild HP phenotype based on the available literature (Yilmaz et al., 2000). Given her mild HP has never escalated to a level requiring treatment (<360umol/L), it does not adequately explain her microcephaly, nor her severe developmental delay, which are now being investigated through genomic techniques. Maternal PKU has been excluded.

## Discussion

### Intronic variants and genotype-phenotype correlation

The most well-characterized intronic variants are those that affect the canonical donor and acceptor splice sites (Ellingford et al., 2022). These four nucleotides are highly conserved, and unnatural variation at these positions can result in retention of intronic material or exon skipping. Canonical splice site variants are therefore likely to have major effects on transcription of the mRNA transcript, and have been classified accordingly in the ACMG criteria (Richards et al., 2015; Abou Tayoun et al., 2018).

Other nucleotide positions in introns are more variable in their conservation, and impacts of genetic variation at these positions depends on multiple factors, as comprehensively summarized recently (Ellingford et al., 2022). The deep intronic *PTS* variant identified in Patient 1 results in the inclusion of a 79 base-pair pseudoexon: however, the majority of transcripts associated with this variant still express normal *PTS* mRNA, predicting significant residual enzyme activity (Chiu et al., 2012). The patient’s other *PTS* variant (c.286G>A) has also been associated with residual PTS activity (Imamura et al., 1999). Identification of the previously reported intronic variant in our patient provided the impetus to cease neurotransmitter supplementation, with no subsequent clinical deterioration. Similarly, Patient 4 has mild hyperphenylalaninaemia that has not required management, in keeping with previous reports of the *PAH* c.969 + 5 variant.

There are other intronic variants where the predicted effect on the phenotype is less clear. For instance, Patient 3 did not present clinically in the neonatal period (similar to the previously reported patient with the same genotype) (Emecen Sanli et al., 2022); however, it is difficult to conclusively determine that she has a mild form of the condition, due to the subsequent recurrent decompensations in childhood. The effects of genetic variation in regions of splice enhancers or splice inhibitors are similarly difficult to predict.

There are additional examples in the literature. Ogino and colleagues reported a patient with OTC deficiency presenting on day one of life, who had undetectable liver OTC enzyme activity, and a hemizygous deep intronic variant (c.540 + 265G>A) was identified (Ogino et al., 2007). However, three other patients with milder forms of OTC deficiency have subsequently been reported with the same variant (Kumar et al., 2021). This suggests that other factors, including physiological stressors and tissue-specific variability in splicing efficiency (and alternative splicing), may be contributing to the phenotypic variability.

### Promoter variants and genotype-phenotype correlation

Promoters, generally located upstream of the 5’ end of the gene, play a crucial role in transcription initiation by recruiting regulatory elements (Maston et al., 2006). These regulatory elements include both general transcription factors (which maintain basal transcriptional activity), and activators (which markedly increase it) (de Vooght et al., 2009). Depending on the degree of interruption of transcription factor binding, sequence variation in the promoter region can impact gene expression.

Promoter variants in IEMs generally appear to be associated with a milder phenotype. Patient 2 is essentially asymptomatic, having been incidentally identified *via* NBS of her unaffected child. The c.-149G>A promoter change in the *SLC22A5* gene has been previously reported as the most common disease-causing variant in a cohort of patients with biochemically suspected primary carnitine deficiency (Ferdinandusse et al., 2019). This variant introduces an upstream out-of-frame translation initiation codon, suppressing translation from the wild-type ATG of *SLC22A5*. This results in reduced (but not absent) OCTN2 protein levels, and concomitantly lower transport activity. The authors reported that no patients carrying this change suffered severe clinical symptoms often associated with primary carnitine deficiency, though it is true that many patients with other variants also do not present clinically (Spiekerkoetter et al., 2003).

We have previously reported three patients with OTC deficiency with late-onset disease, all of whom did not have a pathogenic variant identified by standard molecular analysis of coding regions (Hertzog et al., 2022). Sequencing of the promoter region identified a c.-106C>A variant, which had been reported previously in three other males with late-onset disease (Jang et al., 2018). A dual luciferase assay demonstrated that this promoter variant resulted in 10% of normal gene expression (Han et al., 2022). This was consistent with the milder clinical features of affected males. Provision of timely genetic diagnosis would have assisted with family planning: for many individuals, the diagnostic odyssey can lead families to disengage detrimentally with services (Knerr and Cassiman, 2022), in the false belief that there is no risk of recurrence.

### Strong clinical suspicion of an IEM can guide investigation of non-coding regions

Whilst there is substantial clinical phenotypic variability associated with non-coding variants in IEMs, all of these variants share the commonality of challenges in their detection. There is often poor coverage on whole exome sequencing (WES) beyond the first 100–200 base pairs of the intron. However, in cases where the IEM in question has a highly specific biochemical pattern, this can provide the impetus to assess non-coding regions of the relevant gene when the molecular aetiology has not been identified in a coding region.

In addition, even when a non-coding variant is detected in a proband with a non-specific clinical phenotype (such as non-syndromic intellectual disability or aortopathy), it is rare that such a variant could be classified as pathogenic without genetic evidence (such as segregation) or further functional evidence. Fortunately, in the case of IEMs, there are well-established assays that can confirm specific biochemical phenotypes. This allows laboratories to confidently assess pathogenicity using established criteria, even for those variants that have not been previously reported (Zhang et al., 2020).

With the progressive utilization of WGS as a diagnostic investigation, non-coding variants will become increasingly recognized and, therefore, re-annotated. Many molecularly undiagnosed patients (especially those with a characteristic clinical or biochemical phenotype and a negative trio WES) could benefit from progression to WGS. Additionally, re-analysis of WGS data for pathogenic variants (Costain et al., 2018; Deignan et al., 2019; Robertson et al., 2022) in such cases may be beneficial.

Other “-omics” technologies can also be used in concert with WGS to further characterize non-coding variants. For instance, transcriptomics may be beneficial in quantifying the effects of a genetic change on mRNA transcript production (Bournazos et al., 2022). Proteomics facilitates the assessment of not only whether a genetic change results in decreased (or increased) protein expression, but also whether there are broader changes in the associated biochemical pathway. The benefits of creating infrastructure to accommodate functional validation in the diagnostic pipeline will need to be balanced against the costs of these additional studies (Bournazos et al., 2022).

### Impacts of non-coding variants on management of IEMs

IEMs are often treatable genetic disorders, through various modalities including dietary therapy, medications, enzyme replacement therapy, and organ transplantation (Saudubray et al., 2006). Genotype-phenotype correlation exists in some IEMs, enabling tailoring of therapy to maximize clinical outcome whilst minimizing unnecessary exposure to side effects (Clarke et al., 2019), as seen in Patient 1.

Another important aspect of management is reproductive planning. Patients 1, 3, and 4 all now have molecular diagnoses which can be used by their families for prenatal testing (if they wish). In the case of Patient 4 in particular, the milder genotype may actually provide more confidence not to pursue prenatal testing for what is essentially a biochemical diagnosis without clinical sequelae.

### Future directions of therapy

There are now therapeutics available for non-metabolic conditions, which specifically target non-coding variants, most notably a sub-type of antisense oligonucleotides (AOs) (Kuijper et al., 2021). AOs that target non-coding variants bind to a complementary pre-mRNA strand, and inhibit recognition of this region by the spliceosome through steric hindrance. An established example of this is nusinersen, which treats spinal muscular atrophy by inhibiting splicing of *SMN2*, inducing the inclusion of exon 7 (Wurster and Ludolph, 2018). As a result, more transcript with an intact exon 7 (essentially the same as wild type *SMN1* transcripts) is produced, leading to improved production of full-length SMN protein. The drug has demonstrated clear benefit in improving motor outcome and survival in infants (Finkel et al., 2017); however it also highlights some of the challenges associated with antisense oligonucleotide therapy including high cost, lack of permeability across the blood-brain barrier and requirement of regular dosing (Rinaldi and Wood, 2018).

Similar approaches are being considered for IEMs on the basis of promising *in vitro* studies (Perez et al., 2010), though none of the AOs that promote exon inclusion or pseudoexon exclusion have reached clinical trial (Kuijper et al., 2021). One of the difficulties in drug development in these rare disorders is that such splice variants are also often private mutations, meaning each custom-designed AO therapy may only find use for a handful of patients. As such, there are still considerable challenges to be overcome before these therapies reach routine clinical practice.

## Data Availability

The original contributions presented in the study are included in the article/supplementary material, further inquiries can be directed to the corresponding author.
